# A Secure ZUPT-Aided Indoor Navigation System Using Blockchain in GNSS-Denied Environments

**DOI:** 10.3390/s23146393

**Published:** 2023-07-14

**Authors:** Ali Shakerian, Ali Eghmazi, Justin Goasdoué, René Jr Landry

**Affiliations:** 1 Department of Electrical Engineering, École de Technologie Supérieure, Montréal, QC H3C 1K3, Canada; ali.eghmazi@lassena.etsmtl.ca (A.E.); renejr.landry@etsmtl.ca (R.J.L.); 2 Department of Computer Sciences, Université de Technologie de Compiègne, 60200 Compiegne, France; justin.goasdoue@etu.utc.fr

**Keywords:** indoor navigation, inertial measurement unit (IMU), zero-velocity update (ZUPT), positioning, blockchain, secure, gnss-denied, extended Kalman filter (EKF)

## Abstract

This paper proposes a novel Blockchain-based indoor navigation system that combines a foot-mounted dual-inertial measurement unit (IMU) setup and a zero-velocity update (ZUPT) algorithm for secure and accurate indoor navigation in GNSS-denied environments. The system estimates the user’s position and orientation by fusing the data from two IMUs using an extended Kalman filter (EKF). The ZUPT algorithm is employed to detect and correct the error introduced by sensor drift during zero-velocity intervals, thus enhancing the accuracy of the position estimate. The proposed Low SWaP-C blockchain-based decentralized architecture ensures the security and trustworthiness of the system by providing an immutable and distributed ledger to store and verify the sensor data and navigation solutions. The proposed system is suitable for various indoor navigation applications, including autonomous vehicles, robots, and human tracking. The experimental results provide clear and compelling evidence of the effectiveness of the proposed system in ensuring the integrity, privacy, and security of navigation data through the utilization of blockchain technology. The system exhibits an impressive ability to process more than 680 transactions per second within the Hyperledger-Fabric framework. Furthermore, it demonstrates exceptional accuracy and robustness, with a mean RMSE error of 1.2 m and a peak RMSE of 3.2 during a 20 min test. By eliminating the reliance on external signals or infrastructure, the system offers an innovative, practical, and secure solution for indoor navigation in environments where GNSS signals are unavailable.

## 1. Introduction

A secure and reliable indoor navigation system is essential for a wide range of applications, including emergency response, military operations, and location-based services. However, traditional indoor navigation systems are often vulnerable to privacy breaches and unauthorized access to sensitive location data. To address these concerns, researchers have explored the use of blockchain technology as a tamper-resistant and decentralized platform for data storage and verification. In recent years, there has been growing interest in integrating blockchain technology with zero-velocity updates (ZUPTs)-aided pedestrian inertial navigation systems (INS) for indoor navigation. This integration has the potential to provide a highly secure and trustworthy indoor navigation system that protects users’ privacy and prevents unauthorized access to sensitive location data. This paper proposes a novel secure ZUPT-aided indoor navigation system using blockchain technology, which leverages the strengths of ZUPT-aided pedestrian INS techniques and blockchain technology to provide a highly secure and reliable indoor navigation system.

Zero-velocity updates (ZUPTs) have been widely used in pedestrian inertial navigation systems (INS) for indoor positioning and navigation. A low-cost foot-placed ultra-wideband (UWB) and IMU fusion-based tracking system has been proposed for IoT applications [1]. An enhanced foot-mounted PDR method with adaptive ZUPT and multi-sensor fusion has been developed for seamless pedestrian navigation [2]. Error analysis has been conducted on ZUPT-aided pedestrian INS to identify sources of errors and improve system performance [3]. Improvements in, and evaluations of, zero-velocity detectors have been achieved for foot-mounted INS, including the use of double-threshold zero-velocity updates (ZUPTs) [4] and unscented Kalman filter for accuracy improvement.

Several studies have investigated the performance of ZUPT-aided pedestrian INS and other related systems, including the characterization of foot-mounted ZUPT-aided INSs [5], pseudo-ZUPT re-detection with double-threshold ZUPT for better performance [6], reevaluation of algorithmic basics for ZUPT-based pedestrian navigation [7], scenario-dependent ZUPT-aided pedestrian INS with sensor fusion [8], and smoothing techniques for ZUPT-aided INSs [9]. Other studies have focused on specific aspects of ZUPT-aided pedestrian INS, such as stance-phase detection [10], step detection using foot-mounted permanent magnets [11], estimation errors due to IMU noises [12], and the impact of IMU mounting position on accuracy [13].

The rapid development of wireless communication technology and mobile devices has led to the widespread use of indoor localization and navigation using WiFi sensing. However, this advancement also raises concerns about the privacy and security of users’ personal information and location data [14]. To address this issue, a novel oblivious data sharing scheme utilizing an oblivious transfer protocol has been proposed, which effectively hides location coordinates and protects the privacy of users and servers [14]. Additionally, a string length-based transformation algorithm based on private information retrieval has been integrated into the protocol to reduce communication overhead and ensure the privacy of required keywords [14].

Hyperledger Fabric, a permissioned blockchain platform, offers privacy features and endorsement policies, but there are still privacy risks and attacks associated with disclosing the endorser signature and endorsement policy [15]. To enhance the security of endorsements in Hyperledger Fabric, a framework with a secure endorsement system has been proposed, incorporating an efficient linkable threshold ring signature scheme and a ZK-SNARK set membership proof [15]. The endorsement policy is obfuscated using universal RSA accumulators, and individual membership proofs are batched for efficient validation [15]. While there are several survey papers on indoor positioning methods and the security/privacy of location-based services (LBS), there is a lack of comprehensive research on the security and privacy considerations specifically focused on indoor positioning methods [16]. To bridge this gap, a systematic survey has been conducted, categorizing indoor positioning methods into non-collaborative, collaborative, and other methods, and discussing the security and privacy issues associated with each method [16].

Blockchain technology, particularly Hyperledger Fabric, has gained popularity in various fields, including supply chain and digital identity [17]. Hyperledger Fabric is an open-source platform known for its modularity and versatility, allowing for the establishment of private networks to protect sensitive data. It supports smart contracts in different programming languages, including Solidity, through the Ethereum virtual machine. Its permissioned architecture makes it suitable for domains such as businesses, healthcare, and manufacturing [17]. The continuous evolution of blockchain technology has led to the emergence of new platforms, such as Ethereum and Hyperledger Fabric, which are widely used in industry at present [18]. Different blockchain implementations utilize various consensus protocols, and a brief comparison between Hyperledger Fabric and Ethereum has been provided, considering aspects such as consensus and smart contracts [18].

To ensure the safety of positioning, navigation, and timing (PNT) in satellite navigation systems, a secure PNT system with hybrid physical principles has been proposed, focusing on the augmentations of the BeiDou satellite system (BDS) [19]. This comprehensive PNT infrastructure encompasses deep space and deep ocean, combining BDS and various other technologies. Resilient PNT applications, intelligent PNT services, and key technologies are discussed as future research directions [19]. With the widespread use of Internet of Things (IoT) technology, managing and protecting sensitive private data in data-sharing has become a critical issue [20]. A Hyperledger Fabric blockchain-based secure data-transfer scheme for enterprises in the Industrial Internet of Things (IIOT) has been proposed, utilizing the InterPlanetary File System (IPFS) network and the Elliptic Curve Digital Signature Algorithm (ECDSA) to ensure privacy protection and efficient data transmission [20]. The scheme outperforms traditional networks and existing blockchain systems in terms of throughput, latency, and system overhead [20]. The indoor navigation of unmanned aerial vehicles (UAVs) in GPS-denied areas requires accurate positioning techniques [21]. To improve the accuracy of visual inertial odometry (VIO) in UAV indoor navigation, an integration of ultra-wideband (UWB) positioning and VIO has been proposed, which effectively addresses the limitations of each individual system [21]. The proposed technique achieves centimeter-level accuracy and real-time performance for UAV indoor navigation applications [21].

Hyperledger Fabric Private Blockchain Network (HFPBN) architecture has gained attention due to its potential in various domains [22]. In the case of Vehicular Ad-hoc Networks (VANETs), an architecture for an HFPBN has been explored, considering components such as the ordering service, membership service, and endorsement policy [22]. The architecture provides secure and efficient data sharing and consensus mechanisms for VANETs, ensuring reliable and tamper-proof operations [22]. In addition to the architecture, the block size is a crucial parameter affecting the performance of a Hyperledger Fabric Private Blockchain Network (HFPBN). The impact of block size on performance parameters such as transaction throughput, confirmation time, and resource utilization has been investigated. The findings provide insights into ways to optimizing the block size to achieve a better performance in HFPBNs [22]. Wearable-based collaborative indoor positioning systems (CIPSs) require secure and reliable authentication protocols [23]. A decentralized attribute-based authentication (ABA) protocol has been proposed, which allows for users to access location-based services (LBS) securely and anonymously, leveraging wearable devices and blockchain technology. The protocol ensures the privacy, integrity, and non-repudiation of user attributes, enabling trusted and collaborative positioning in CIPSs [23].

Blockchain technology has demonstrated potential in improving supply chain management and traceability [24]. Focusing on the coffee supply chain industry, an analysis has been conducted on the application of blockchain, specifically Hyperledger Fabric, in enhancing transparency, accountability, and sustainability in the coffee supply chain. The proposed implementation utilizing smart contracts and immutable ledgers provide a tamper-proof and trustworthy system for tracking coffee products [24]. Multi-robot collaboration in industrial applications, such as inventory management, requires efficient coordination and localization mechanisms [25]. To address this challenge, a framework has been proposed, integrating Hyperledger Fabric blockchain, UWB localization, and a multi-robot system for inventory management [25]. The framework ensures reliable data sharing, secure transactions, and accurate localization, enhancing the efficiency of inventory management in industrial settings [25].

In recent years, there has been a growing need for secure and reliable indoor navigation systems that can protect users’ privacy and prevent unauthorized access to location data. Several studies have explored the use of blockchain technology to provide a tamper-resistant and decentralized platform for data storage and verification [26,27,28]. However, the integration of blockchain technology with ZUPT-aided pedestrian INS techniques has not been extensively studied. A recent study proposed a symmetrical-net method that uses adaptive zero-velocity detection for ZUPT-aided pedestrian navigation, which improves the accuracy of the system [6]. Therefore, there is a need for further research on the development of secure and reliable indoor navigation systems that can meet the growing demand for privacy-preserving and trustworthy location-based services, while leveraging the strengths of blockchain technology and advanced ZUPT-aided pedestrian INS techniques.

To address this need, this study proposes a secure ZUPT-aided indoor navigation system using blockchain technology. The system aims to provide reliable and accurate indoor navigation while ensuring the security and privacy of users’ location data. The proposed system leverages the strengths of ZUPT-aided pedestrian INS techniques and blockchain technology to develop a tamper-resistant and decentralized platform for data storage and verification. This system is expected to be particularly useful in scenarios where privacy and security are critical, such as healthcare facilities, financial institutions, and military facilities. The proposed system can also be extended to other applications that require accurate and secure indoor navigation, such as robotics, autonomous vehicles, and smart homes.

## 2. Materials and Methods

This section describes the methodology and algorithms used in this project in two sections. In the first sections, the focus will be on the mechanization of a foot-mounted IMU, which involves the integration of ZUPT, inertial measurement unit (IMU) measurements, and an extended Kalman filter (EKF) algorithm. The ZUPT equations and their mathematical background will be discussed, along with the mathematical principles of the IMU. Additionally, the EKF algorithm will be explained, and its role in improving the accuracy of the foot-mounted IMU will be highlighted. These methods will be used to estimate the position and orientation of a pedestrian in indoor environments, with the goal of achieving higher accuracy than traditional methods. In the second section, the methodology regarding Blockchain will be explained. This will include a discussion on the fundamentals of blockchain technology, Hyperledger-Fabric, and its role in securing data in the context of the pedestrian positioning system described in the first section.

### 2.1. Indoor Navigation

This section aims to provide a brief overview of the methodology employed in the navigation section. The discussion will encompass a brief analysis of the ZUPT equations and their mathematical foundation, as well as the fundamental mathematical principles underpinning the IMU. Additionally, a brief explanation of the EKF algorithm will be presented.

Furthermore, the developed server and Ground Control Station (GCS) will be elucidated, with a particular emphasis on their functionality in the context of this research. This will facilitate a deeper understanding of the various components involved in the navigation system and their roles in enabling accurate and reliable navigation.

#### 2.1.1. Inertial Measurement Unit (IMU)

To discuss the selection of an appropriate IMU for the pedestrian motion tracking system, various factors such as size, weight, power, and cost (SWaPC) were taken into consideration. In this regard, we have chosen to use two MPU-20948 from InvenSense. This is a MEMS-based IMU that combines the 3-axis accelerometer, 3-axis gyro and the 3-axis magnetometer, making it a low-cost 9-DOF IMU. One of the advantages of this IMU is its small size and low power consumption. Table 1 illustrates the specifications of this IMU, which provides inertial data at a sampling rate of 100 Hz.

**Figure 1:**
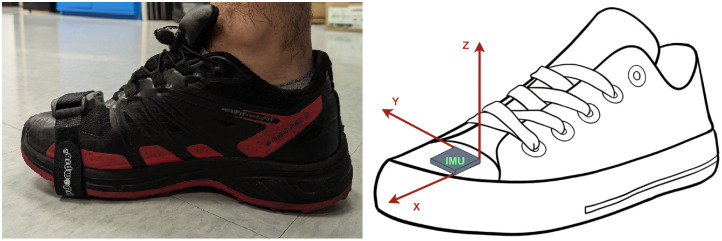
Overview of the placement and alignment of the ibNav IMU on the users foot.

The IMU was placed on the forefoot in this study, which has been proven to be a more favorable mounting position than the heel in ZUPT-aided pedestrian inertial navigation to capture the sequential nature of pedestrian motion and utilize error-reduction techniques such as zero-velocity update (ZUPT) during foot stances/swings. This is attributed to several advantages, including a longer stance phase, reduced shock levels, and decreased velocity uncertainty during the stance phase [13]. We assume that the user is walking along the global x-axis, and the sensor’s local (body) coordinates and global coordinates are shown in Figure 1. Our algorithm is developed irrespective of the exact position and orientation of the INS on the user’s shoe.

Aggarwal, in [29], provides the simplified mechanization equations for IMU. In these equations, *n* and *b* represent the navigation frame and sensor body frame, respectively. The specific force vector of the accelerometer is represented by $f^{b}$, and the angular rate vector of the gyroscope is represented by $w_{ib}^{b}$. The position vector and velocity vector in the earth-centered earth-fixed frame are represented by $p^{n}$ and $v^{n}$. The transformation matrix from the body frame to the navigation frame, as a function of attitude components, is represented by $C_{b}^{n}$. The skew-symmetric matrices of the gyroscope-based angular rate vector are represented by $w_{ib}^{b}X$. Finally, the gravity vector in the navigation frame is represented by $g^{n}$.

(1) $$\left[\begin{array}{c} p^{n}\\ v^{n}\\ C_{b}^{n} \end{array}\right]=\int \left[\begin{array}{c} v^{n}\\ C_{b}^{n}f^{b}+g^{n}\\ C_{n}^{b}\left(w_{ib}^{b}X\right) \end{array}\right]$$

In this study, we make the assumption that the initial velocity is zero, and it is further assumed that the initial position is already known. The starting angle is then calculated using the IMU, which operates based on the following principles:

(2) $$\left\{\begin{array}{c} \phi =\arctan\frac{g_{y}^{b}}{q_{z}^{b}}\\ \psi =\arctan\frac{g_{x}^{b}}{\sqrt{{\left(g_{y}^{b}\right)}^{2}+{\left(g_{z}^{b}\right)}^{2}}} \end{array}\right.$$

The precise determination of attitude angles is crucial. In this regard, the gravity components $g_{x}^{b}$, $g_{y}^{b}$, and $g_{z}^{b}$ in the body frame can be utilized to determine the roll angle ϕ and pitch angle ψ of the attitude. It is noteworthy that the accelerometer readings predominantly reflect the gravity components when the velocity is zero, indicating that they remain independent of linear acceleration. As a result, the horizontal attitude angles can be reliably estimated from the accelerometer readings. Following the determination of the horizontal angles, the magnetometer can be aligned with the horizontal position to facilitate the precise calculation of the heading.

(3) $$\left\{\begin{array}{c} m_{x}=m_{x}^{b}\cdot \cos\psi +m_{y}^{b}\cdot \sin\phi \cdot \sin\psi +\sin\psi \cdot \cos\phi \cdot m_{z}^{b}\\ m_{y}=m_{y}^{b}\cdot \cos\phi -\sin\phi \cdot m_{z}^{b} \end{array}\right.$$

where $m_{x}$ and $m_{y}$ stand for the components of magnetic measurements in the horizontal direction, and $m_{b}^{x}$, $m_{y}^{b}$, and $m_{z}^{b}$ are the magnetometer measurements in the sensor frame. The heading angle can be calculated as follows:

(4) $$\alpha =\arctan\frac{m_{x}}{m_{y}}$$

**Figure 2:**
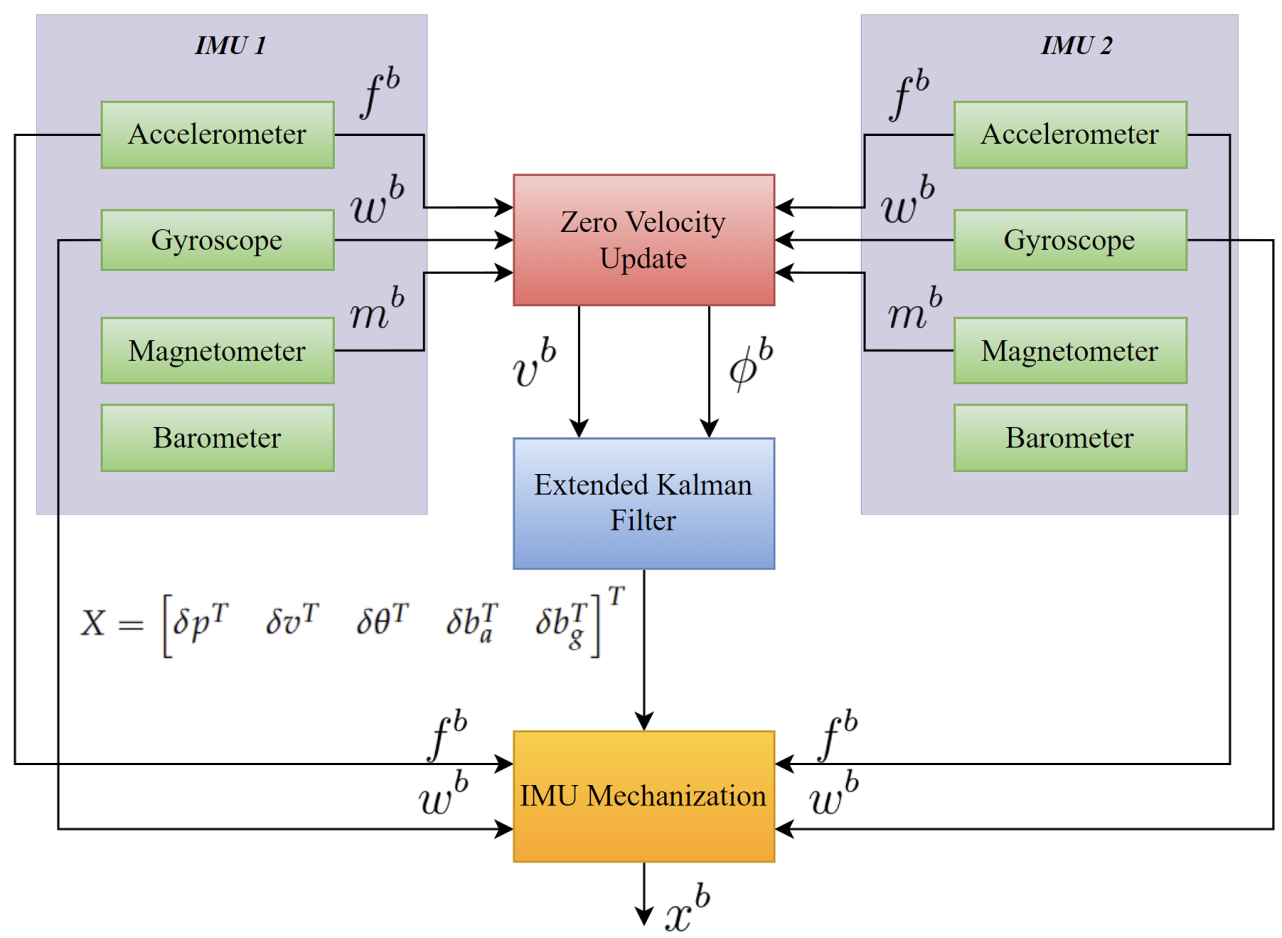
Overview of the Inertial Navigation Architecture with ZUPT and EKF for Dual-IMU MEMS-Based Setup.

Figure 2 provides a high-level overview of the architecture in the system used for dual-IMU ZUPT-aided navigation.

#### 2.1.2. Zero Velocity Update (ZUPT)

The zero-velocity update (ZUPT) is a technique used in navigation systems to improve the accuracy of position estimates by detecting when a pedestrian or vehicle is stationary and correcting any drift errors in the sensor measurements during that stationary period The zero-velocity update (ZUPT) method, which uses an inertial measurement unit (IMU), can greatly increase the accuracy of location estimates during stationary periods, where the pedestrian’s or vehicle’s velocity and angular velocity are close to zero. The ZUPT technique compares the magnitude of acceleration and angular velocity measurements to a threshold value in order to solve a binary hypothesis testing issue. The position estimate is updated with zero-velocity information if the magnitude is less than the threshold number, at which point the pedestrian or vehicle is assumed to be stationary. This is crucial since sensor drift can cause substantial positioning errors, necessitating the use of zero-velocity data. While ZUPT is effective in most walking scenarios, it may not be suitable for activities such as running, jumping, and climbing due to its threshold-based approach [4]. Using the collected data, the ZUPT algorithm performs initial alignment, monitors acceleration to detect stationary periods, estimates gyroscope bias, corrects for bias in angular velocity measurements, integrates position estimates to correct for drift, and repeats the process for subsequent motion intervals. By minimizing drift, utilizing stationary periods, suppressing noise, and offering real-time updates for accurate position estimate, this approach increases accuracy.

#### 2.1.3. Extended Kalman Filter (EKF)

To address the issue of drift in navigation outcomes caused by the nonlinear system model used in the mechanization algorithm, we employ the extended Kalman filter (EKF). Unlike the standard Kalman filter, the EKF is capable of dealing with nonlinear models by approximating them through linearization around the current state estimate. The EKF can handle nonlinear models, making it well-suited to fusing additional update information and reducing drift in the mechanization algorithm’s navigation results. The mechanization algorithm’s navigation outcomes can drift apart quickly over time because of inertial sensor noise and system model inaccuracies. To alleviate this divergence, the extended Kalman filter (EKF) is typically employed to blend additional update data [2]. To implement the ZUPT in the EKF, it is crucial to accurately identify the stationary state of the user’s foot during the stance phase. If this is not done correctly, the velocity estimate error would drastically increase, leading to a quadratic growth in the position estimate error and state error vector over time [11]. The state error vector is stated as follows:

(5) $$X={\left[\begin{array}{ccccc} \delta p^{T} & \delta v^{T} & \delta \theta^{T} & \delta b_{a}^{T} & \delta b_{g}^{T} \end{array}\right]}^{T}$$

where δp, δv, and δθ are the errors of the location, velocity, and attitude errors in 1. The bias errors of the accelerometer and gyroscope are represented by $\delta b_{a}$ and $\delta b_{g}$, respectively. The system model in discrete form can be shown as follows:

(6) $$\left\{\begin{array}{c} X_{k}^{-}=\Phi_{k}X_{k-1}+G_{k}W_{k-1}\\ P_{k}^{-}=\Phi_{k}P_{k-1}\Phi_{k}^{T}+G_{k}Q_{k-1}G_{k}^{T} \end{array}\right.$$

The terms $X_{k}^{-}$ and $P_{k}^{-}$ denote the anticipated state and its corresponding covariance matrix. The matrix $\Phi_{k}$ represents the transition of the state from the previous epoch (k−1) to the current epoch (*k*). $G_{k}$ is the matrix that determines the effect of state noise. $W_{k-1}$ and $Q_{k-1}$ are the vector and matrix, respectively, which describe the system noise. The matrix $F_{k}$ can be calculated using the state differential formula in Equation (1) and the bias error model. Finally, the discretized state transition matrix can be obtained through the following process [2]:

(7) $$\Phi_{k}=I+F_{k}\cdot T_{s}=\left[\begin{array}{ccccc} I_{3} & I_{3}\cdot T_{s} & 0_{3} & 0_{3} & 0_{3}\\ 0_{3} & I_{3} & \left(f^{b}\times \right)\cdot T_{s} & C_{b}^{n}\cdot T_{s} & 0_{3}\\ 0_{3} & 0_{3} & I_{3} & 0_{3} & -C_{b}^{n}\cdot T_{s}\\ 0_{3} & 0_{3} & 0_{3} & I_{3} & 0_{3}\\ 0_{3} & 0_{3} & 0_{3} & 0_{3} & I_{3} \end{array}\right]$$

The discretization time interval of the state equation is denoted by $T_{s}$. The matrix $f^{b}\times$ is a skew-symmetric matrix formed from a specific fore vector. The state noise gain matrix $G_{k}$ and system noise covariance matrix $Q_{k-1}$ are defined with explicit detail as [2]:

(8) $$G=\left[\begin{array}{cccc} 0_{3} & 0_{3} & 0_{3} & 0_{3}\\ C_{b}^{n}\cdot T_{s} & 0_{3} & 0_{3} & 0_{3}\\ 0_{3} & -C_{b}^{n}\cdot T_{s} & 0_{3} & 0_{3}\\ 0_{3} & 0_{3} & I_{3}\cdot T_{s} & 0_{3}\\ 0_{3} & 0_{3} & 0_{3} & I_{3}\cdot T_{s} \end{array}\right]$$

(9) $$Q=\left[\begin{array}{cccc} I_{3}\cdot w_{f}^{b} & 0_{3} & 0_{3} & 0_{3}\\ 0_{3} & I_{3}\cdot w_{\omega }^{b} & 0_{3} & 0_{3}\\ 0_{3} & 0_{3} & I_{3}\cdot {\tilde{w}}_{f}^{b} & 0_{3}\\ 0_{3} & 0_{3} & 0_{3} & I_{3}\cdot {\tilde{w}}_{\omega }^{b} \end{array}\right]$$

The terms $I_{3}$ and $0_{3}$ represent a 3 × 3 diagonal matrix with ones on the main diagonal and a matrix filled with zeros, respectively. $w_{f}^{b}$ and $w_{\omega }^{b}$ denote the noise present in the measurements taken from accelerometers and gyroscopes. Similarly, ${\tilde{w}}_{f}^{b}$ and ${\tilde{w}}_{\omega }^{b}$ refer to the noise that influences the accelerometers and gyroscopes [29].

#### 2.1.4. Hardware and Connection Setup

The ibNav 6.1 sensor is specifically designed for indoor navigation and is equipped with a dual-IMU setup, providing accurate and reliable navigation data, as discussed in the previous sections. To collect and transmit the navigation data, we utilized the Message Queuing Telemetry Transport (MQTT) protocol, which is a lightweight and efficient communication protocol commonly used in IoT applications.

The collected data were transmitted to a Raspberry Pi 4, which served as a gateway device, via the Wi-Fi wlan0 connection. To ensure the reliability and stability of the data transmission, we used a separate Wi-Fi dongle and established a connection via MQTT wlan1 to transmit the processed data to our server, named RiCF, and Blockchain for storage and analysis purposes. The use of blockchain technology in this context provides an added layer of security and transparency, ensuring the integrity of the data using Proof Of Location (POL). The POL mechanism involves cross-referencing the data from multiple sources to verify the device’s location within the indoor environment. This added layer of validation helped to eliminate any potential errors or inaccuracies caused by data being transmitted from a different location.

Ground Control Station (GCS) plays a critical role in the operation of the system, providing a centralized platform for data visualization and the monitoring of system performance. With the GCS, the platform can receive and display data from RiCF and Blockchain in real-time, allowing the system to quickly identify and analyze any issues or anomalies in the data. The GCS provided a user-friendly graphical interface that enabled us to easily visualize the data and gain insights into the performance of the system. Additionally, the GCS allows users to remotely control the system and adjust various parameters, such as data transmission rates and processing algorithms, to optimize the performance of the system. Overall, the GCS is an essential component of the system, providing a powerful tool for real-time data analysis and system management.

**Figure 3:**
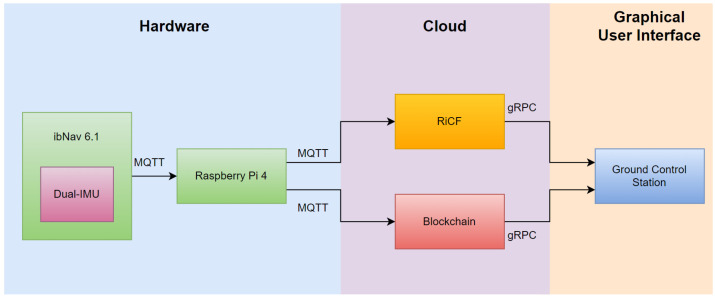
A comprehensive data flow architecture setup.

Figure 3 illustrates the system architecture, providing a clear and concise overview of the components and their interactions.

#### 2.1.5. RiCF Server

**Figure 4:**
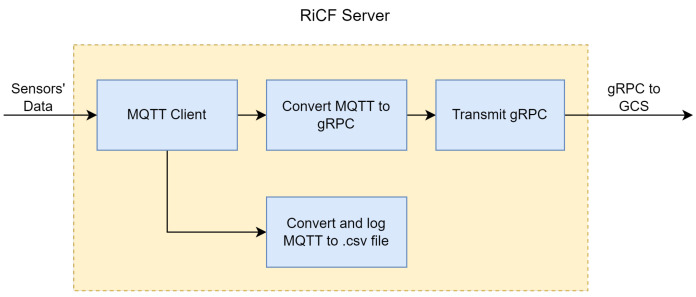
A general architecture of RiCF.

RiCF (RPNT-ibNav Control framework) is a cross-platform Python server that is intended to consolidate and simplify communication between all Resilient Positioning and Timing (RPNT)-ibNav components including ibNav 6.1 sensors and Raspberry Pi and, on the other interface, transmit it to the Ground Control Station (GCS). The RiCF server centralizes communication between such different components with diverse applications for compatibility. The MQTT Protocol is the communication link between RiCF and the components while RiCF and Ground Control Station communicate by Google Remote Procedure Call (gRPC). In contrast to MQTT, gRPC is developed to support several languages, improve application compatibility, and use Google’s Protocol Buffer to specify structured data for communication. When using gRPC, server, and client communication is seamless, enabling clients to call server functions directly as if they were on a local object. On the contrary, the MQTT protocol is lightweight and ideal for the ibNav system used by users with Raspberry Pi, Lidar, and inertial sensors. However, it is not suitable for a server-based system since it is a messaging protocol without a payload format specification, and interoperability between applications depends on prior agreement on a format. Additionally, MQTT is a publish/subscribe protocol, and the broker sends messages to all subscribers of a particular topic without differentiating clients. To provide a more detailed explanation of data processing in RiCF, Figure 4 is presented for reference. The users’ data, collected from inertial sensors, are transmitted via MQTT through the local network using the WiFi bridge, received by the RiCF server, and logged into a corresponding CSV file for post-processing purposes. Simultaneously, the messages are processed and formatted to be utilized as gRPC messages. Real-time data streaming is then transmitted to GCS for visualization and demonstration.

#### 2.1.6. Ground Control Station (GCS)

**Figure 5:**
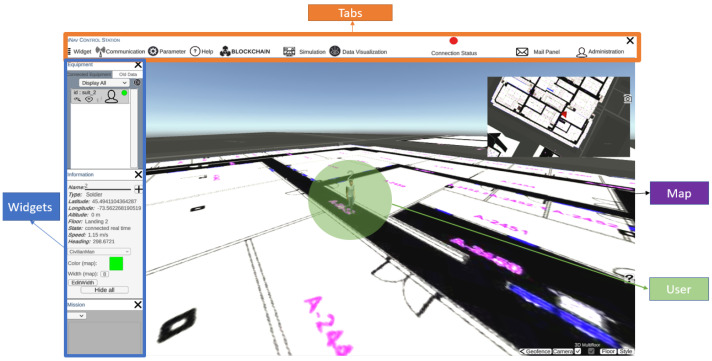
A global view of the Ground Control Station.

The Ground Control Station displays information to users to identify and direct activities, including human-controlled UAVs. It receives and displays data from the IMU setup worn by users. GCS has an easy and practical application design. In order to optimize the design of the GCS, it is important to prioritize the main functionalities to be easily accessible to the operator. Less frequently used functionalities should also be included but hidden from view to minimize distractions and maintain the operator’s focus. This approach can enhance the overall usability and effectiveness of the GCS. Additionally, it is essential for a GCS to be equipped with the ability to automatically open relevant panels upon receiving emergency messages, to ensure prompt notification to the operator without requiring any manual action. The communication between the client application and server, facilitated by the gRPC protocol, allows the application to make requests to the server, such as retrieving information or reading a file. This communication also enables the application to access the various features available on the server. The server sends data to the application following a specific structure, where a message can consist of multiple elements. The gRPC protocol implemented in the application allows for it to receive and identify each element of the message sent by the server. The global view of the GCS is presented in Figure 5. GCS offers a wide range of beneficial applications, including its capacity for facilitating collaborative indoor navigation. With the integration of diverse tools for navigating indoors and drones, users can explore uncharted territories within this field using the GCS platform. Despite its limited development in collaborative indoor navigation methods, GCS remains prepared to provide full support for their implementation while offering exciting possibilities for advancing knowledge.

GCS has several tabs and functionalities to control and visualize the navigation for the user. The widgets tab includes the equipment, alerts, errors, information and mission windows. Communication functionality is to start or end a real-time navigation test, although playback tests are available for developers. The parameters tab allows the user to connect to the desired IP address. The blockchain tab sends requests to the IPFS to request stored data in the Blockchain. A notable feature in GCS is the trajectory feature that shows the selected user’s route on the map. GCS can display the trajectory of equipment based on the floor selected by the operator on the map, especially when the equipment is indoors. Moreover, the application provides a multi-floor reference event in 2D, where only the trajectories on the appropriate floor are displayed. Multiple trajectories may be displayed if the equipment returns to the same floor multiple times or only a single trajectory if it travels to one floor and does not return. The GCS platform boasts a potential ability to generate an environment map through the utilization of Simultaneous Localization and Mapping (SLAM) algorithms alongside appropriate sensors, including LiDAR and camera. However, this particular feature has yet to be implemented, and thus may serve as a promising avenue for future research and development.

### 2.2. An Overview of Blockchain Technology

The concept of blockchain was first introduced in 2008 by a person using the pseudonym “Satoshi Namakato”. The innovation was applied to create the world’s first cryptocurrency, Bitcoin, in 2009 [30]. Since then, blockchain has become a buzzword in various industries, and its use cases have expanded beyond cryptocurrency. Blockchain is a decentralized ledger built on consensus principles to have a verifiable, append-only chained data system of transactions. Because of its decentralized architecture, blockchain makes it easy to store and upgrade the data, rendering blockchain a perfect architecture for ensuring distributed transactions, such as avionics networks, among all participants in a trustless setting. Appearing from the intelligence assets, a smart contract enables a team to accomplish agreements among parties via a blockchain network [31]. Based on how much confidence is needed in the network, blockchain confidentiality will be chosen for the network. Due to its short transaction latency and high throughput, the permissioned blockchain Fabric is better suited for handling IoT interactions [32].

#### 2.2.1. Developing Blockchain Solutions by Hyperledger-Fabric

Hyperledger Fabric, launched in 2015 as part of the Hyperledger project by the Linux Foundation, is a highly modular and adaptable blockchain platform. It provides a permissioned network in which individuals must be identified and networks must be permitted. Hyperledger Fabric prioritizes the privacy and secrecy of business transactions and related data while focusing on a high transaction throughput speed and low transaction confirmation latency. The possibility of malicious code being inserted through a smart contract is reduced in this permissioned setting. Hyperledger Fabric, like other blockchain technologies, employs a ledger and smart contracts to provide a way for participants to manage their transactions [33].

Hyperledger Fabric is an open-source blockchain-based distributed ledger targeting enterprises, with smart contracts providing a high degree of trust. It serves as a foundation for developing distributed ledger systems, with a modular structure that is adaptable and scalable, guaranteeing strong privacy and resilience [34].

Hyperledger Fabric’s shared ledger consists of the global state and transaction log, with each participant having a copy. Smart contracts, called chaincode, interact with the ledger’s world state and can be written in various programming languages. Hyperledger Fabric caters to different privacy requirements, supporting both highly private networks using channels and more open networks. Transactions in Hyperledger Fabric are recorded in the order they occur, and various consensus methods, such as PBFT and mining, can be chosen based on network relationships and structures [33].

**Figure 6:**
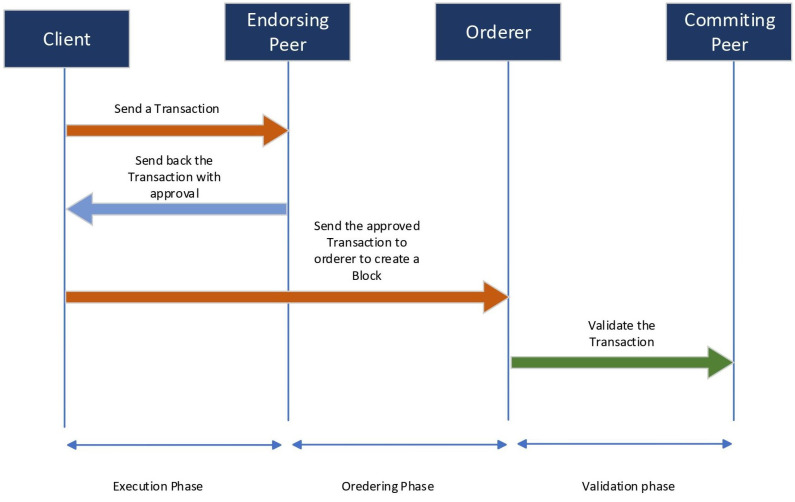
Transaction Flow in Hyperledger-Fabric.

The Hyperledger-Fabric blockchain comprises critical components such as domain, organizations, peers, orderers, and certificate authorities. Physical separation of the blockchain network is achieved using organization. Nodes validate and store transactions under an execute–order–validate transaction flow, and they are classified as a client, peer, and ordering nodes depending on their processing and storage capacity. Peers maintain the blockchain ledger, execute the chain code (or smart-contracts), and commit transactions to the global state. Orderer nodes provide communication channels and generate new blocks based on the consensus mechanism, while the certificate authority confirms ownership [32,34]. Figure 6 illustrates the Transaction flow in Hyperledger-Fabric.

Hyperledger Fabric implements various security measures including ledger immutability, consensus mechanisms, membership services provider (MSP), secure communication, code isolation, and auditing to address vulnerabilities and enhance system resilience against attacks. These measures ensure tamper-proof transaction records, prevent unauthorized data modifications, manage identities and access control, encrypt communication, isolate code, and enable monitoring and auditing for enhanced security. Regular updates and adherence to best practices are essential for maintaining a secure Hyperledger Fabric network.

#### 2.2.2. Hyperledger-Fabric Deployment

In this deployment, the Hyperledger Fabric Blockchain was leveraged as a secure and reliable database to store data generated by Raspberry Pi devices. To ensure the authenticity and integrity of the devices, each was assigned a unique public–private key pair, employing RSA 2048-bit encryption. The fabric Software Development Kit (SDK) played a vital role in this process by providing the necessary resources and libraries to establish communication between the devices and the Hyperledger Fabric Blockchain. Through the utilization of JSON Web Tokens (JWT) protected with RSA-based encryption, the SDK verified the authentication of the devices, guaranteeing secure and trusted communication.

**Figure 7:**
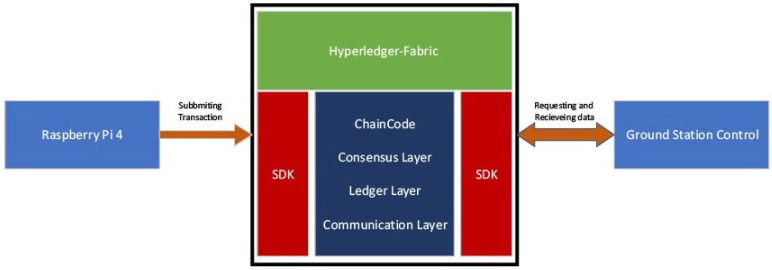
Overview of Hyperledger-Fabric Deployment.

Once the authentication mechanisms were in place, the Raspberry Pi devices could securely interact with the Hyperledger Fabric Blockchain. They were capable of submitting data transactions to the blockchain network, which were subsequently stored in the distributed ledger. Figure 7 provides a concise overview of the deployment process for Hyperledger-Fabric. The fabric SDK facilitated this interaction, ensuring that transactions were properly verified and certified before being included in the ledger, thereby maintaining the integrity of the data.

## 3. Results and Discussion

In accordance with the methodology used in this study, the following part will be divided into two separate subsections, each concentrating on a different element of the analysis. The first subsection will examine the system’s navigation performance. The emphasis of the second subsection will be on the interpretation and efficacy of the Blockchain used in the system. Building on the previous subsection’s analysis, this part will look at how Blockchain can improve the system’s effectiveness, security, and dependability. Furthermore, it will assess the effectiveness of the Blockchain implementation in meeting the system’s particular requirements and address any possible limitations or challenges that may have emerged during the analysis.

### 3.1. Indoor Navigation Performance Analysis

In order to evaluate the performance of our system in real-time, we conducted a series of indoor tests in École de technologie supérieure (ÉTS). The tests varied in duration from 10 to 30 min and involved providing the Ground Control Station (GCS) with a reference trajectory and an initial position. The GCS then displayed the real-time position of the user throughout the test, while an ibNav 6.1 sensor was mounted on the user’s foot to collect the necessary data. Table 2 illustrates the selected test configuration chosen for our tests. It should be noted that data collected from the IMUs transmit to the Raspberry Pi through MQTT using Mosquitto with the frequency of 100 Hz on port 1883.

The use of an ibNav 6.1 sensor allowed us to collect navigation data, which we subsequently used to calculate the final trajectory and Root Mean Square Error (RMSE) for each test. The RMSE metric provided us with a measure of the deviation between the reference trajectory and the actual trajectory, allowing us to evaluate the system’s accuracy.

**Figure 8:**
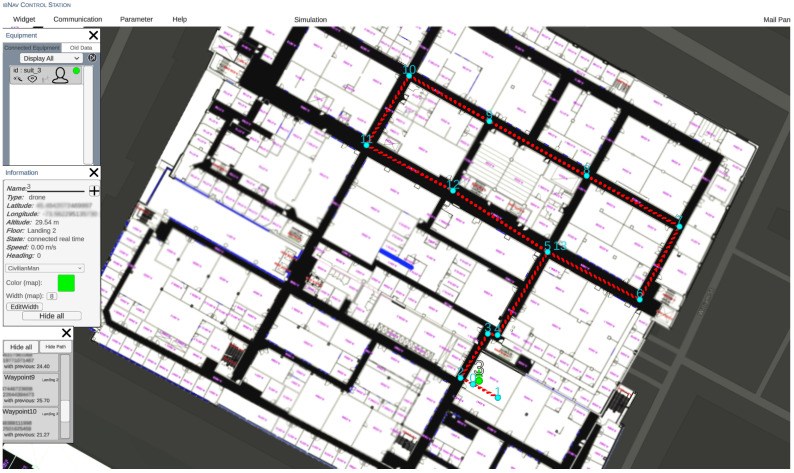
Visualizing a Sample Test Trajectory on GCS Platform: Map of ÉTS with Reference Path.

In order to analyze the navigation performance of the system, a reference trajectory is given to GCS, as seen in Figure 8. While the reference trajectory is not mandatory when performing an indoor test, it is necessary to have a reference in order to analyze the error and performance of the proposed platform.

**Figure 9:**
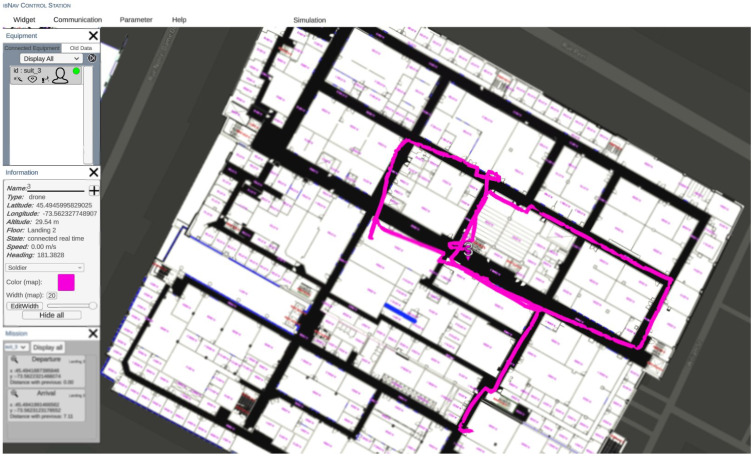
Visualizing a sample test trajectory on GCS platform: map of ÉTS with reference path.

**Figure 10:**
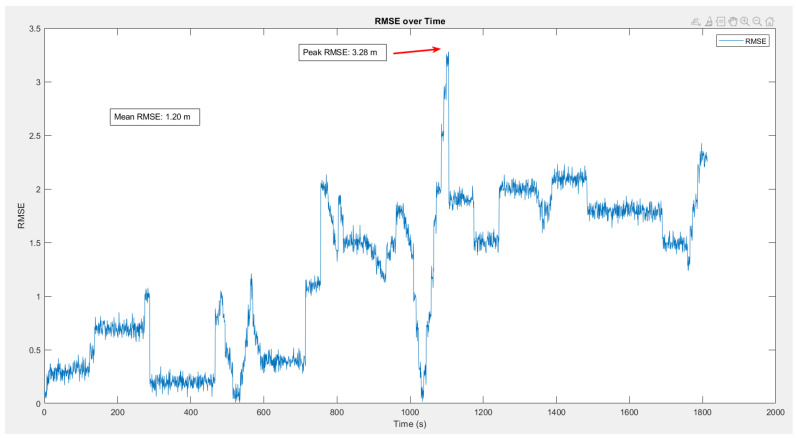
RMSE analysis over a sample test of 30 min.

After saving the reference points to the GCS, we started the test by launching the realtime connection. The test took 30 min and 12 s. The results of the tests are presented in Figure 9, and demonstrate the system’s high level of accuracy and reliability. This result is shown in the GCS platform as a user interface. However to precisely calculate the RMSE error of the test, data collected from the ibNav 6.1 are saved in a CSV file with a corresponding CSV file of the reference trajectory. Then, MATLAB software was used to calculate the RMSE of the whole test. Figure 10 illustrates the RMSE over the duration of the test. As shown in this figure, the mean RMSE is 1.2 m and the peak RMSE is 3.28 m.

A number of significant insights were gleaned from an in-depth examination of the collected data, shedding light on the system’s performance under real-time situations. The results unambiguously indicate the system’s astonishing capacity to track the user’s position in real-time and navigate the environment with exceptional precision.

It is commonly acknowledged that heuristic drift and numerous environmental conditions can provide substantial obstacles, hindering the performance of navigation systems that rely on Inertial Measurement Units (IMUs). Nonetheless, the technology under examination overcomes these challenges and achieves an outstanding performance that exceeds current restrictions.

However, it is important to recognize the experiment’s scope, which is mostly focused on easy walking settings, ignoring the incorporation of more difficult movements such as running and jumping. As a result, the results of the experiment should be evaluated in this context. The lack of a comprehensive analysis of these complicated scenarios requires additional research to determine the system’s abilities and potential limitations in a variety of dynamic settings.

Delving deeper into the examination of sophisticated scenarios involving complex actions, such as running and jumping, undoubtedly has tremendous promise for future research endeavors. This investigation would not only improve our understanding of the system’s behaviour under stress, but would also open the door to new possibilities and enhancements, assuring the system’s continual evolution and optimisation in real-world applications.

### 3.2. Blockchain Performance Analysis in Hyperledger Fabric

Following the successful deployment, the stored data within the Hyperledger Fabric Blockchain became accessible for analysis and utilization. The Ground Control Station (GCS) played a pivotal role in this stage as it served as the central interface for accessing and retrieving data from the Blockchain. Functioning as a centralized administration and tracking system for the Raspberry Pi devices, the GCS provided users with the necessary tools to manage and handle their activities effectively. To enhance the user experience and enable efficient data analysis, a Graphical User Interface (GUI) was developed. The GUI offered a visually appealing representation of the acquired data, incorporating various graphical elements and real-time updates. This user-friendly interface facilitated easy interpretation and evaluation of the data collected from the Raspberry Pi devices. Users could gain valuable insights and make informed decisions based on the knowledge derived from the Hyperledger Fabric Blockchain.

In summary, this deployment showcased the successful integration of the Hyperledger Fabric Blockchain’s robustness and reliability with the data collection abilities of Raspberry Pi devices. The utilization of the fabric SDK and the implementation of robust authentication procedures ensured secure and seamless communication between the devices and the blockchain network. The Ground Control Station (GCS) provided a comprehensive interface for data access and management, while the Graphical User Interface (GUI) enhanced data visualization and analysis, empowering users to leverage the insights provided by the Hyperledger Fabric Blockchain for informed decision-making.

In order to measure the performance of the platform, we used Hyperledger Caliper to measure the performance of Blockchain. Hyperledger Caliper is a free and open-source benchmarking tool for blockchain networks. It enables users to evaluate and quantify the performance of various blockchain implementations based on a predefined set of use cases. The program is extremely flexible, allowing customers to customize their testing scenarios to their exact requirements. Hyperledger Caliper is a vital tool for blockchain developers and system administrators looking to maximize the efficiency of their blockchain networks, thanks to its user-friendly interface and extensive functionality. The benchmark test was run on a hyperledger fabric blockchain network with three organizations, and each organization has one peer and three orderers. The test was run on a Ubuntu machine with 16 GB RAM and an Intel(R) Core(TM) i7-9750H CPU @ 2.60GHz. In order to eliminate any biases from our performance test, we performed three rigorous tests to ensure the stability and reliability of the results. Table 3 depicts the performance of a blockchain network as measured by the Hyperledger Calliper tool for writing and reading transactions in the tests. The analytical findings reveal the lowest and highest Transactions Per Second (TPS) attained during the trial. The investigation specifically examined the TPS for both the creation of a new transaction and for reading a transaction from the blockchain network.

**Figure 11:**
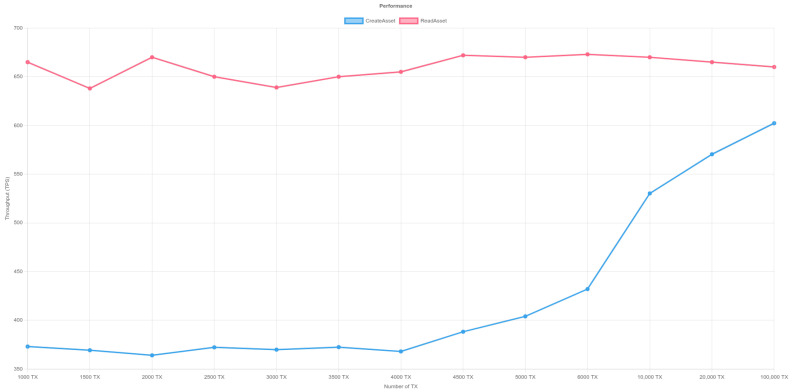
Performance of Hyperledger-Fabric- Test 1. Y-axis represents throughput (TPS) and X-axis shows the transaction number. The red line illustrates the read asset function while the blue line represents the create asset.

**Figure 12:**
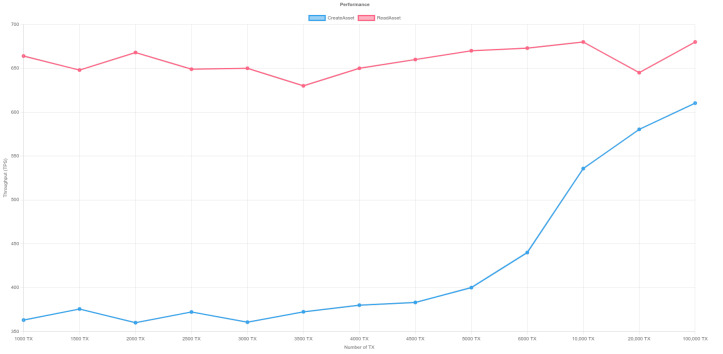
Performance of Hyperledger-Fabric- Test 2. Y-axis represents throughput (TPS) and X-axis shows the transaction number. The red line illustrates the read asset function while the blue line represents the create asset.

**Figure 13:**
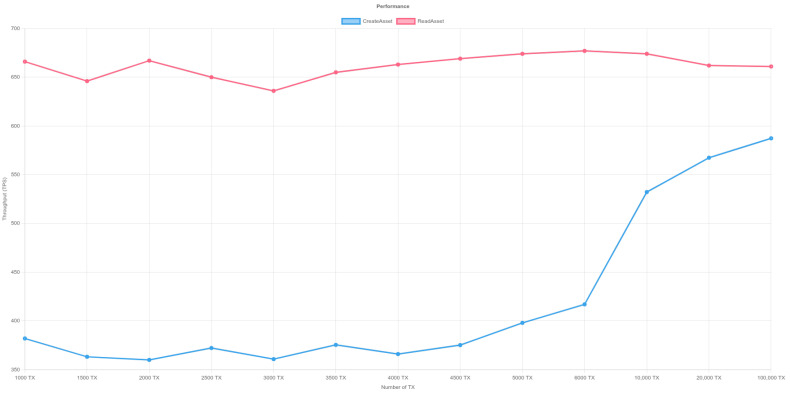
Performance of Hyperledger-Fabric- Test 3. Y-axis represents throughput (TPS) and X-axis shows the transaction number. The red line illustrates the read asset function while the blue line represents the create asset.

The lowest recorded TPS for creating a new transaction is 360 and the highest recorded TPS is 610.3. As the number of transactions grows, so does the TPS. However, as the quantity of transactions rises, the success percentage of accepting transactions falls. Figure 11, Figure 12 and Figure 13 illustrate the outcomes of reading transactions from the blockchain network. Interestingly, the TPS figures for reading transactions are relatively constant, ranging from 630 to 680. This means that the blockchain network is more optimized for reading transactions than it is for producing new ones. Furthermore, when the number of transactions rises, the success percentage of accepting transactions for reading remains roughly consistent. This indicates that the network is capable of processing a high amount of read requests without affecting the accuracy of the data being retrieved.

## 4. Conclusions

To address the challenges of providing accurate and secure indoor navigation in GNSS-denied environments, this paper proposes a novel blockchain-based indoor navigation system that integrates a foot-mounted dual-inertial measurement unit (IMU) setup and a zero-velocity update (ZUPT) algorithm. The suggested system uses an extended Kalman filter (EKF) to combine data from two IMUs and the ZUPT method to rectify the error caused by sensor drift during zero-velocity periods. By offering an immutable and distributed database to store and validate sensor data and navigation solutions, the blockchain-based design guarantees the system’s security and reliability. Based on tests conducted in École de technologie supérieure (ÉTS) university department of electrical engineering, the experimental results show that the proposed system achieves high accuracy and robustness in indoor environments, with a mean RMSE error of 1.2 m and a peak RMSE of 3.28. Without depending on exterior signals or infrastructure, the suggested system provides a feasible and safe solution for interior guidance in a variety of uses such as driverless cars, robots, and human monitoring. Because of the system’s ability to provide precise and secure interior navigation in GNSS-denied environments, it is a hopeful option for future indoor navigation systems that require reliable and secure navigation. When considering the scalability of the proposed platform, it is important to note that the system can be readily implemented in any environment with a WiFi connection. This is facilitated by the utilization of MQTT and gRPC protocols, which are compatible with WiFi technology. The flexibility of WiFi connectivity enables the system to be deployed in various settings, providing convenience and accessibility for users. In terms of sensor durability, the ibnav sensor, as mentioned in the paper, can sustain approximately 90 min of continuous usage. This lifespan provides a reasonable timeframe for typical applications and usage scenarios. However, it is worth noting that the duration may vary depending on specific use cases and sensor settings. Conducting further research or experimentation to evaluate the sensor’s performance under different conditions would enhance our understanding of its operational limits and potential battery life improvements.

The integration of LiDAR in the future has enormous potential to improve system performance. Not only may introducing LiDAR into the system boost its abilities, but it can also lead to more accurate and dependable outcomes. Furthermore, integrating cameras with Visual SLAM can improve indoor navigation by providing a thorough and real-time understanding of the surroundings. To ensure the system’s robustness, it should be rigorously tested in a variety of weather situations. The system’s performance in different temperatures may be fine-tuned to work effectively and reliably in a variety of conditions, maximizing its utility and applicability. Furthermore, including augmented reality (AR) can significantly improve the system’s performance. AR can overlay useful information over the user’s real-world perspective, such as instructions, points of interest, or safety alerts. This integration improves not only navigation but also the overall user experience, making it more intuitive and engaging.

## Figures and Tables

**Table 1:** Specifications of the ICM-20948.

Specifications	Accelerometer	Gyroscope	Magnetometer	Barometer
Measurement Range	±2 g, ±4 g, ±8 g, ±16 g	±250 dps, ±500 dps, ±1000 dps, ±2000 dps	±4900 μT	260–1260 hPa
Sensitivity	16,384 LSB/g	131 LSB/dps	0.15 μT/LSB	0.01 hPa/LSB
Noise Density	200 μg/$\sqrt{\mathrm{Hz}}$	20 mdps/$\sqrt{\mathrm{Hz}}$	0.15 μT/$\sqrt{\mathrm{Hz}}$	0.01 hPa/$\sqrt{\mathrm{Hz}}$
Output Data Rate (ODR)	4 Hz–1.12 kHz	4 Hz–8 kHz	4–100 Hz	1–200 Hz
Current Consumption (Operating)	450 μA	1.2 mA	90 μA	0.9 mA

**Table 2:** Selected Test Configurations for the ICM-20948.

Specifications	Accelerometer	Gyroscope	Magnetometer	Barometer
Measurment Range	8 g	1000 dps	4900 μT	1000 hPa
Low Pass Filter	Enabled	Enabled	Disabled	Disabled
Output Data Rate (ODR)	1.125 kHz	1.125 kHz	100 Hz	100 Hz

**Table 3:** Comparison of three sets of tests using hyperledger caliper.

Test Number	Min TPS	Max TPS	Avg TPS
Test 1- Read	638	673	660.846
Test 1- Create	364	602.3	411.746
Test 2- Read	630	680	656.692
Test 2- Create	360	610.3	428.623
Test 3- Read	636	677	661.077
Test 3- Create	360	587.3	433.777

## Data Availability

Not applicable.
